# Spatial epidemiology of human schistosomiasis in Africa: risk models, transmission dynamics and control^☆^

**DOI:** 10.1016/j.trstmh.2006.08.004

**Published:** 2007-01

**Authors:** Simon Brooker

**Affiliations:** Department of Infectious and Tropical Diseases, London School of Hygiene and Tropical Medicine, Keppel Street, London WC1E 7HT, UK

**Keywords:** Schistosomiasis, Spatial epidemiology, Geographical information systems, Remote sensing, Geostatistics, Control, Africa

## Abstract

This paper reviews recent studies on the spatial epidemiology of human schistosomiasis in Africa. The integrated use of geographical information systems, remote sensing and geostatistics has provided new insights into the ecology and epidemiology of schistosomiasis at a variety of spatial scales. Because large-scale patterns of transmission are influenced by climatic conditions, an increasing number of studies have used remotely sensed environmental data to predict spatial distributions, most recently using Bayesian methods of inference. Such data-driven approaches allow for a more rational implementation of intervention strategies across the continent. It is suggested that improved incorporation of transmission dynamics into spatial models and assessment of uncertainties inherent in data and modelling approaches represent important future research directions.

## 1 Introduction

Spatial epidemiology is the study of the spatial variation in patterns of infection and disease and of the causes and consequences of such heterogeneity. The scientific study of the spatial epidemiology of schistosomiasis and other helminths has been greatly enhanced by the use of geographical information systems (GIS) and remote sensing (RS) over the past 20 years. The former has enabled data to be georeferenced, stored, extracted, integrated in new ways and displayed by the user (Robinson, 2000), whilst the latter has provided high-resolution data on climate and land cover features (Hay et al., 2006). Since Cross et al. first used Landsat™ satellite data to predict the occurrence of schistosomiasis in the Philippines and the Caribbean (Cross and Bailey, 1984, Cross et al., 1984), an increasing number of studies have employed GIS/RS to predict the distribution of schistosomiasis on the basis of associations between infection and large-scale environmental variables (Brooker et al., 2001, Brooker et al., 2002a, Brooker et al., 2002b, Clements et al., 2006a, Clements et al., 2006b, Malone et al., 1994, Malone et al., 2001, Moodley et al., 2003, Raso et al., 2005, Raso et al., 2006). The emphasis of most of these studies has been to iteratively develop more accurate and statistically robust risk models, increasingly adopting a Bayesian inferential platform. Less emphasis has been given to assessing either the uncertainties inherent in geographical data (Agumya and Hunter, 2002) or the practical application of models in the context of large-scale control activities (Brooker et al., 2002c).

Here, I provide a brief review of how recent field and modelling studies have improved our understanding of the spatial epidemiology of schistosomiasis in Africa, but also attempt to illustrate the relevance of this research in the targeting of large-scale schistosomiasis control programmes as well as to identify future research directions. My focus is schistosomiasis in Africa since this is where both the disease burden and the need for control remain greatest. For applications of GIS/RS for the study of the epidemiology and control of Asian schistosomiasis, the reader is referred to a recent excellent review by Yang et al. (2005).

## 2 Spatial heterogeneity of schistosomiasis

Schistosomiasis in Africa is due predominately to *Schistosoma mansoni*, which causes intestinal schistosomiasis, and *S. haematobium*, which causes urinary schistosomiasis. The geographical distribution of schistosomiasis across the continent was first comprehensively mapped nearly 20 years ago through a synthesis of historical records, documents and published reports, including hospital-based data (Doumenge et al., 1987). However, this traditional cartographic approach has the disadvantage that the derived maps cannot be updated easily and it is therefore unable to reflect recent epidemiological trends. For instance, changes in transmission have occurred as a consequence of (i) man-made ecological changes such as the construction of large dams and irrigation schemes and (ii) the successful implementation of control (Fenwick et al., 2006). A more recent project has employed GIS to develop a comprehensive database of human helminth infection in Africa and provides district-level spatial descriptions across the continent, thereby identifying areas for which further information is required (Brooker et al., 2000). Building upon this work, the WHO has recently established a global helminth databank that also includes data on coverage of anthelmintic treatment programmes (www.who.int/wormcontrol/databank/en/).

**Figure 1:**
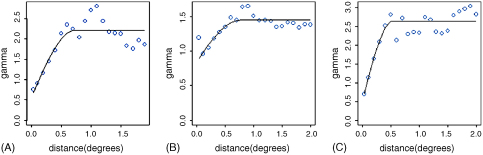
Patterns of the spatial structure of *Schistosoma mansoni* in (A) Cameroon, (B) Mali and (C) Uganda. Omnidirectional semivariograms and best-fitted lines of exponential spatial models for de-trended log prevalence data based on Generalized Additive Model (GAM) residuals of longitude, latitude, rainfall, elevation and maximum land surface temperature. Based on parasitological data from Brooker et al. (2002a), Traore et al. (1998) and Kabatereine et al. (2004). Note: at the equator, 1 decimal degree equates to approximately 120 km.

Aside from the obvious benefits of data capture and visualisation, the integrated use of GIS and RS, coupled with geostatistical techniques, has allowed the robust quantification of spatial heterogeneity in schistosome infection patterns. In particular, geostatistics can determine whether patterns are due to either random stochastic processes and/or variability in the estimated prevalence because of small population sizes for some units, or are, in fact, caused by specific variables such as environmental heterogeneity (Bailey and Gatrell, 1995). At the community level, a useful tool to quantify the spatial structure of infection patterns is the semivariogram, which describes the spatial correlation of observations and is computed by measuring the mean-squared difference of pairs of observations that are separated by the same distance (Chiles and Delfiner, 1999). Figure 1 presents semivariograms for the prevalence of *S. mansoni* in three different transmission settings across Africa. In each setting, the variogram exhibits considerable spatial structure up to a range of 70 km, even after removing large-scale trend effects. After this distance, there was an apparent lack of spatial correlation or structure. These results therefore suggest that spatial factors in addition to climatic factors are influencing the spatial distribution of *S. mansoni* prevalence at distances of up to 50 km. This knowledge can help to determine the spatial scale at which various geographical factors influence spatial distributions and to identify optimal sampling strategies.

Improved accuracy of geographical positioning systems has facilitated the investigation of household patterns both of schistosome infection (Clennon et al., 2004, Utzinger et al., 2003) and associated morbidity, including hepatomegaly and fibrosis (Booth et al., 2004). Because patterns of infection and disease vary strongly by demographic variables, such as age and sex, it is important that analysis takes adequate account of these factors as well as the spatial correlation in the data. Bayesian spatial modelling can provide smoothed estimates of the intensity of schistosome infection while adjusting for spatial correlation and the highly aggregated distributions of egg counts as well as individual-level covariates such as age and sex (Brooker et al., 2006a).

Recent theoretical work on the persistence of infectious diseases using transmission models indicates that persistence typically decreases with increasing spatial heterogeneity (Hagenaars et al., 2004, Woolhouse et al., 1998). The significance of spatial heterogeneity of schistosomiasis to the overall transmission and the effectiveness of different intervention strategies is poorly understood at present (Gurarie and King, 2005). An important first step to improving our understanding is better quantification of observed heterogeneities across a variety of spatial scales, and is an area that merits further study.

## 3 Landscape epidemiology of schistosomiasis

Schistosomiasis occurs across the African continent in numerous geographic landscapes of varied characteristics, in which specific climatic, physical and human characteristics influence the intensity of transmission. Through a knowledge of the characteristics necessary for the transmission of infection, it is possible to understand and predict the spatial and temporal distribution of infection. The idea of using landscape features to understand the spatial heterogeneity of infectious diseases extends back to the 1930s through the work of the Russian parasitologist Evgenii Pavlovsky who coined the phrase ‘landscape epidemiology’.

The schistosome parasite requires a molluscan intermediate host in which to undergo development, and freshwater snails form an essential component in the lifecycle of schistosomiasis. This ties transmission to landscapes where people and snails come together at the same water habitat. Numerous factors act to determine the rate of transmission in a given location. These include biotic and abiotic features, such as climatic, physical and chemical factors that affect the survival and development of schistosome parasites and snail host populations (Sturrock, 1993), as well as socioeconomic and behavioural characteristics of the human community such as water contact behaviour and the adequacy of water and sanitation, which affect the frequency and intensity of exposure to infected water (Bundy and Blumenthal, 1990).

Here, it is worth highlighting an important feature of the transmission dynamics of schistosomiasis relevant to understanding spatial distributions. Overall transmission success depends crucially on the establishment, survival and fecundity of adult schistosomes in the human host, and depends less on the survival and fecundity of the two free-living aquatic stages, the miracidia and cercaria, and of the infected snail hosts (Anderson, 1987). This is because the lifespan of adult worms is substantially longer (3–6 years) than those of either infected snail hosts (weeks) or free-living stages (hours). For this reason, the most significant determinants of the intensity of transmission are changes in water contact patterns through improved water and sanitation and health education, or changes in parasite mortality through the implementation of population-based chemotherapy. However, if these factors remain unchanged, then the rate of parasite establishment and hence the patterns of schistosomiasis are primarily determined by the distribution and abundance of its intermediate hosts, freshwater snails.

The most important determinants of the population dynamics of snails are temperature and rainfall (reviewed in Sturrock, 1993). The optimal temperature for snail development and survival is around 25 °C. Above 30 °C snail mortality increases, and thermal death occurs at 40 °C. However, snails are less sensitive to low temperatures than schistosome parasites in snails. Uninfected snails can therefore be found in high altitude areas of endemic countries where low temperatures inhibit larval development in snails. Several studies have demonstrated marked spatial and temporal heterogeneity in snail population dynamics owing to fluctuations in rainfall (Sturrock, 1993). However, it is difficult to quantify precisely the spatial relationships between rainfall and snail population dynamics and schistosome transmission since the effect of rainfall varies according to snail species and geographical location. Moreover, seasonal fluctuations in snail dynamics are of limited significance to overall parasite transmission since adult schistosomes typically have a longer lifespan relative to such seasonal fluctuations (Anderson, 1987).

Delineation of the climatic limits of schistosome transmission at continental scales has been enhanced by the integrated use of GIS and satellite sensor data (Brooker, 2002, Brooker and Michael, 2000, Kabatereine et al., 2004, Malone et al., 2001, Moodley et al., 2003). However, such broad-scale patterns belie the tremendous complexity and variability in transmission between different foci and even within the same focus. This focal distribution is suggested to reflect the small-scale distribution of habitats suitable for snail species and the multiple factors that determine habitat suitability (Woolhouse et al., 1991, Woolhouse et al., 1998). These include physical and chemical factors such as pH, vegetation and water velocity (Sturrock, 1993), and man-made ecological changes such as the construction of large dams and irrigation schemes (Jordan and Webbe, 1993). Genetic differences in interspecific and intraspecific intermediate host–parasite interactions and infectivity may also play a role (Rollinson et al., 2001), although this aspect remains poorly understood. Despite these small-scale heterogeneities and generative mechanisms, it is suggested that large-scale environmental and climatic factors influence the broader-scale patterns of parasite transmission, such that climate-based risk maps can be developed.

## 4 Risk mapping of schistosomiasis

In initial risk mapping studies, predictions were largely based either on simple threshold analysis (Malone et al., 2001) or on traditional regression modelling (Brooker et al., 2001, Brooker et al., 2002a, Brooker et al., 2002b) to predict the presence/absence of infection or the prevalence above a certain threshold. In such regression modelling, however, spatial correlation in infection and environmental data, as illustrated in Figure 1, is ignored. This omission can underestimate the standard errors of the covariate coefficient and lead to erroneous inference of the importance of some covariates in explaining variation in infection patterns (Chiles and Delfiner, 1999).

Bayesian methods of inference, which offer a flexible and robust modelling approach, have increasingly been applied in spatio-epidemiological studies (Diggle et al., 1998) and have several implicit advantages over traditional, frequentist regression approaches. First, they can readily take into account the spatial variability in the epidemiological and environmental data, whereby spatial processes are assumed to be normally distributed according to a cross-correlation function, typically defined by the semivariogram of the spatial process (Chiles and Delfiner, 1999). Second, the Bayesian paradigm can account for model uncertainty by assuming that the model itself, as the parameter values, varies as a random quantity (Clyde and George, 2004). In this approach, model uncertainty has a straightforward probabilistic interpretation.

The first application of Bayesian geostatistics to the risk mapping of schistosomiasis in Africa was undertaken by Raso et al. (2005) who investigated the demographic, socioeconomic and environmental risk factors explaining the geographical distribution of *S. mansoni* infection in a small area of western Côte d’Ivoire. An important result arising from their work was that small-scale spatial variation in age, sex and socioeconomic status showed a stronger influence on the geographical variation of infection patterns compared with the environmental covariates investigated. More recently, Clements et al. (2006a) have developed Bayesian geostatistical models to predict the spatial distributions of *S. haematobium* and *S. mansoni* infections across a large area of northwest Tanzania. Their approach highlighted important species-specific differences in observed spatial correlations, with correlations occurring over greater distances for *S. haematobium* than for *S. mansoni*. In addition, maps of prediction error highlighted which areas need further investigation if maps are to be uniformly reliable.

**Figure 2:**
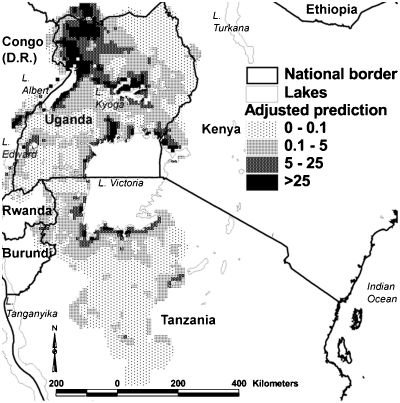
Predicted intensity of infection (eggs/g faeces) with *Schistosoma mansoni* in East Africa, adjusted for environmental covariates (distance to perennial water body and land surface temperature) based on a Bayesian geostatistical negative binomial model (modified from Clements et al., 2006b).

Attempts to predict the distribution of schistosomiasis have until now been based on the use of point prevalence data rather than estimates of infection intensity. This is because prevalence is an easily collected and readily available indicator. However, intensity is an important determinant both of transmission dynamics and morbidity, the two indicators of greatest relevance to the design of disease control strategies (Anderson, 1987). Modelling of infection intensity is none the less complicated by the fact that parasite distributions are highly aggregated within communities. The negative binomial distribution provides a good empirical description of observed distributions of egg counts, allowing the degree of aggregation to be defined by a single parameter, *k*, an inverse measure of aggregation. To predict the spatial distribution of intensity of *S. mansoni* infection in East Africa, Clements et al. (2006b) have recently developed a Bayesian geostatistical model that explicitly incorporates a negative binomial distribution. Results identified the role of environmental risk factors in explaining spatial heterogeneity in infection intensity and showed how these factors can be used to develop a predictive map (Figure 2). The linkage of such spatial models of intensity with mathematical models of schistosome transmission dynamics (Chan et al., 1996), as recently demonstrated for malaria transmission models (Gemperli et al., 2006), offers an exciting prospect of maps that can estimate the effectiveness of different control strategies through space. This is clearly an area that needs and deserves more careful investigation.

Three additional features of risk mapping merit further scientific study. First, the importance of different demographic, socioeconomic and environmental risk factors will differ according to the spatial scale of investigation. Over broad scales, risk of helminth infection is associated with climatic factors (Brooker, 2002, Brooker et al., 2006b). At local scales of a few kilometres, transmission risk is related to the spatial heterogeneity in human demography and socioeconomic status (Raso et al., 2005). At the community level, human behavioural and snail ecological factors are important (Woolhouse et al., 1991, Woolhouse et al., 1998). Studies investigating the importance of different risk factors at varying spatial scales and their relative importance in transmission dynamics are therefore clearly necessary.

Second, the precise extent of the spatial correlation in data will be influenced by local characteristics and will therefore be expected to differ in different parts of a geographical region (Chiles and Delfiner, 1999). Modelling of such non-stationary spatial processes has received scant attention, and the few available studies have focused on methods that decompose the spatial domain into disjoint regions and assume a separate stationary process in each region, but where the data are assumed independent across regions (Gemperli, 2003, Kim et al., 2005). It may also be possible to model the spatial field using a single non-stationary process akin to ARIMA (auto-regressive integrated moving average) models of time series analysis.

Third, risk models have considered the epidemiology of parasite species in isolation, whereas in fact the majority of human infections typically involve multiple species. Raso et al. (2006) have recently combined demographic, environmental and socioeconomic data and Bayesian geostatistics to assess risk factors and spatial variation of *S. mansoni*–hookworm co-infection on a local scale in Côte d’Ivoire. This approach permits the robust prediction of co-infection, thereby helping to guide integrated disease control programmes (Lammie et al., 2006). Spatial modelling of co-infection and its use as part of a fuller consideration of polyparasitism in humans is long overdue.

## 5 Spatial targeting of schistosomiasis control

In a previous review (Brooker, 2002), I noted that, despite progress in the use of GIS and RS to understand better the epidemiology and ecology of schistosomiasis in Africa, there had been few attempts to apply the developed methodology to actual control scenarios. Since then, an increasing number of international initiatives have been established that aim to reduce the disease burden caused by schistosomiasis and other helminth infections. For instance, we have seen the establishment of the Schistosomiasis Control Initiative (SCI), which is currently supporting six countries in sub-Saharan Africa to implement national control programmes (Garba et al., 2006, Kabatereine et al., 2006). In each programme, there is a need to define national policy and to prioritise areas where intervention is most needed and will produce the greatest benefit.

**Figure 3:**
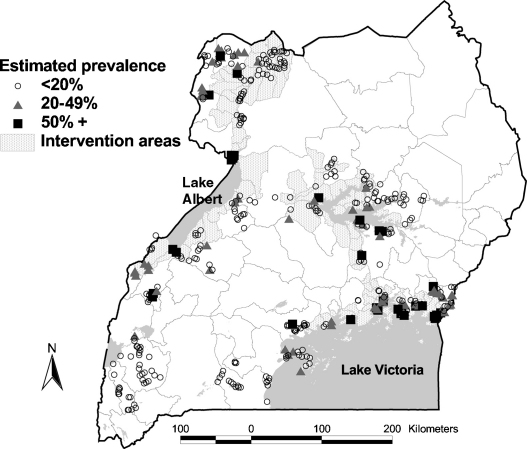
Distribution of *Schistosoma mansoni* in Uganda in 2006. Data are based on the results of a rapid mapping survey conducted in 31 districts using the Lot Quality Assurance Sampling (LQAS) technique.

In Uganda, where *S. mansoni* is widespread, GIS and RS have been employed to classify the country according to different treatment strategies. Initial geographical analysis indicated an absence of transmission in areas where total annual rainfall was <850 mm or altitude was >1400 m (Kabatereine et al., 2004). These areas were subsequently set aside without the need for additional surveys (Brooker et al., 2004). It was further shown that prevalence consistently exceeded 50% in areas within 5 km of Lakes Victoria and Albert, and thus these areas warranted mass treatment without the need for further surveys. Outside these two ecological areas, where smaller rivers and water bodies are numerous, rapid parasitological mapping of communities at local scales has been undertaken using Lot Quality Assurance Sampling (LQAS) to target control finely (Brooker et al., 2005) (Figure 3). Here, four teams, comprised of one supervisor, two technicians and a driver, visited different regions of the country and sampled eight schools per day over 3 days per district, at an overall cost of US$21 182. The purpose of the exercise was to classify schools according to different prevalence thresholds (<20%, 20–50% and >50%) and the results were presented and discussed at a national planning workshop and used to target control more effectively at subdistrict levels.

In Tanzania, Bayesian risk mapping has been used to determine subdistricts warranting mass treatment (Clements et al., 2006a). Here, separate risk models for *S. haematobium* and *S. mansoni* were combined to make a single intervention map (as the treatment programme and its use of praziquantel makes no distinction between the two schistosome species), consisting of contours that equated to a prevalence of *S. haematobium* or *S. mansoni* of 10% and 50%. Outside of Africa, GIS/RS applications have successfully guided the control of schistosome infection among cattle, water buffaloes and humans in China (Yang et al., 2005), as well as aiding the assessment and monitoring of ecological changes in relation to climate change and large water resource development projects. The above experiences illustrate how GIS/RS can provide a data-driven approach to the implementation of intervention strategies, thereby enhancing resource allocation.

Notwithstanding this potential, it should be recognised that risk mapping is one of a number of alternative approaches that may be employed to target schistosomiasis control. For example, a morbidity questionnaire can effectively be used to identify communities with a high prevalence of *S. haematobium* (Lengeler et al., 2002), and LQAS has been used successfully to target control for *S. mansoni* at local scales (Brooker et al., 2005). However, I would argue that risk mapping should be used, in the first instance, to exclude low-risk areas (Brooker et al., 2002b) and that rapid mapping techniques, such as questionnaires and LQAS, should subsequently be used to target control locally. An important area of future research is to assess the cost effectiveness of alternative methods to identify communities requiring mass treatment with praziquantel, as has recently been demonstrated for the rapid mapping of *S. mansoni* (Brooker et al., 2005).

## 6 Spatial transmission dynamics and uncertainties

Over the past three decades, our understanding of the transmission dynamics of helminths has been greatly aided by the development of mathematical models (Anderson and May, 1991). Research on the transmission dynamics of schistosome infection and disease has traditionally focused on changes in disease patterns with time or, in practice, with age (Chan et al., 1996). The spatial dimension is also particularly important for helminth transmission dynamics, but until recently few studies have addressed this issue (Gurarie and King, 2005). By contrast, the importance of spatial heterogeneity to microparasite transmission has received increasing attention (Ferguson et al., 2005, Keeling et al., 2001, Smith et al., 2002). For instance, spatiotemporally explicit models of disease dynamics were used to investigate the spread and control of the foot and mouth disease outbreak in the UK (Keeling et al., 2001) and, more recently, to investigate the spread of pandemic influenza in Southeast Asia (Ferguson et al., 2005). It remains surprising that this feature has not been addressed for macroparasites given the observed associations between environmental variables and transmission processes. In practical terms, such studies would require detailed quantitative data, both in space and time, and necessitate sophisticated mathematical models.

Uncertainty in models and data might also dissuade us from developing spatially explicit models of schistosome transmission. Uncertainty pervades throughout spatial epidemiology, manifesting in all stages of data collection and analysis, from parasitological diagnosis, satellite sensor and population data to the modelling methods themselves (Agumya and Hunter, 2002, Atkinson and Graham, 2006). Such uncertainty is particularly apparent in Africa where data are often extremely sparse but where there exists a strong need for rational decision-making to help maximise cost-effective resource allocation. Uncertain information is easily distorted when quantified and expressed in the form of a map, and this in turn can lead to disease control and resource allocation decisions that are misleading; therefore, the analysis and management of uncertainty is of vital importance.

A promising platform to deal with uncertainty in situations of near-ignorance is the Dempster–Shaffer Theory (Dempster, 1966), a generalisation of Bayesian theory that is thought to represent uncertainty better in near-ignorance situations (Luo and Caselton, 1997). Such a framework allows the inclusion of expert, although subjective, opinion in model development as well as empirical model parameters and therefore offers greater flexibility than Bayesian approaches when quantifying uncertain information and more closely reflects its consequences (Luo and Caselton, 1997). Another approach able to tackle uncertainty includes Fuzzy set theory, which can deal with and assess the extent to which a given condition is true, and has previously been used to develop a risk map for stable transmission across Africa (Craig et al., 1999). The future application of such knowledge-driven spatial modelling and uncertainty management has the potential to enhance the use of available information towards rational decision-making in disease control.

## 7 Conclusion

During the last two decades, the use of GIS/RS has provided an invaluable analytical tool to understand better the large-scale determinants of schistosome infection and has developed reliable ways to identify populations for mass treatment. This research has provided clear evidence for the link between spatial patterns of schistosome infection and climatic factors that can be determined by RS technologies. More recent work has explicitly incorporated spatial correlations of infection into risk models and has provided a robust assessment of statistical uncertainty. One of the more interesting aspects of recent modelling studies concerns the role of non-climatic factors in determining spatial distributions, especially at local scales (Raso et al., 2005). Paradoxically, therefore, future research in this area requires detailed field studies on the determinants of infection at multiple spatial scales. A further topic that warrants attention is the integration of, essentially static, spatial predictive models with mathematical models of transmission dynamics, and in particular the investigation of how transmission dynamics varies functionally in relation to environmental heterogeneity. Such work is underway for microparasites (Smith et al., 2002) but similar research is warranted for schistosomiasis. Addressing these questions is not only of academic interest but is also vital to the development of rational control strategies. A final area that merits further attention, and is indeed germane to the long-term and sustainable use of GIS/RS in schistosomiasis control, is the use of the developed approaches in post-intervention settings, but also as part of a suite of mapping approaches to target integrated control activities.

## Conflicts of interest statement

The author has no conflicts of interest concerning the work reported in this paper.
